# Diagnostic Clue in a Neonate with Amniotic Band Sequence

**DOI:** 10.1155/2020/8892492

**Published:** 2020-10-01

**Authors:** Raffaele Falsaperla, Marta Arrabito, Piero Pavone, Valentina Giacchi, Tiziana Timpanaro, Paolo Adamoli

**Affiliations:** ^1^Neonatal Intensive Care, AUO San Marco-Policlinco, University of Catania, Catania, Italy; ^2^Department of Clinical and Experimental Medicine, Section of Pediatrics and Child Neuropsychiatry, University of Catania, Catania, Italy; ^3^U. O. di Pediatria, Ospedale Generale di Zona Moriggia-Pelascini Como, Gravedona ed Uniti, Italy

## Abstract

Amniotic band syndrome (ABS) is a set of congenital malformations that mainly affect the limbs and more rarely the skull, face, chest, and abdomen. Two main hypotheses have been proposed to explain the nature of the disorder: an intrinsic and extrinsic factor. We report a newborn with ABS presenting with several malformations involving both hands and foot. In this case, the malformative event localized at the hands and right foot without involvement of any other internal organs and is asymmetric which leads us to suppose the extrinsic factor as cause of the ABS.

## 1. Introduction

Amniotic band syndrome (ABS), also named amniotic band sequence, is a congenital disorder presenting with fetal anomalies associated with fetal placental fibrous bands that may cause disruptions, deformations, or malformations [1]. The clinical presentation of ABS is wide and ranges from relatively minor and isolated malformations to multiple malformations affecting several bodily areas, in some cases with fatal outcomes. Head, face, or internal organs may be affected; in most cases, however, the arms and legs are the most commonly affected and typically present with an asymmetric distribution [2]. The etiology of ABS remains unknown. Two main hypotheses have been proposed to explain the nature of the disorder: intrinsic factors acting within the fetus and extrinsic factors that act on the fetus externally [3]. The disorder has been suggested to be caused by both of these factors. The incidence varies from 1 in 1,200 to 1 in 15,000 live births [4]. Here, we report a newborn with ABS presenting with several malformations involving both hands and one foot. In this case, the malformation event was localized and asymmetric, suggesting an extrinsic factor as the cause of ABS.

## 2. Case Report

**Figure 1:**
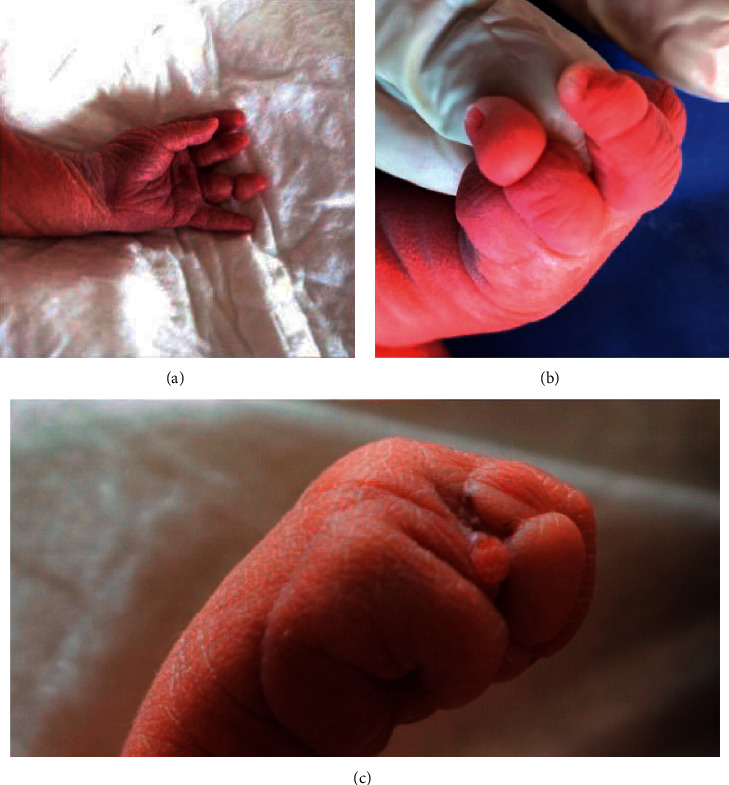
Left hand. Single palmar crease, deep annular sulcus in the fourth finger deep (a); annular sulcus in the fourth finger and interdigital bridges (b); annular sulcus (c).

**Figure 2:**
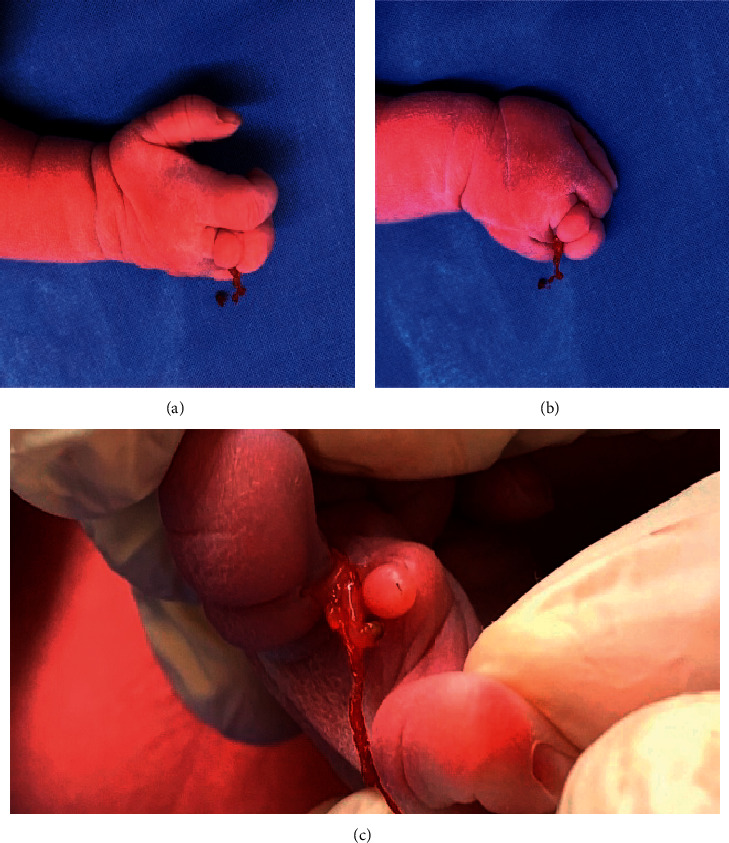
Right hand. Annular furrows on the third finger (a); spontaneous amputation of the fourth finger (b); the amputated finger (c).

**Figure 3:**
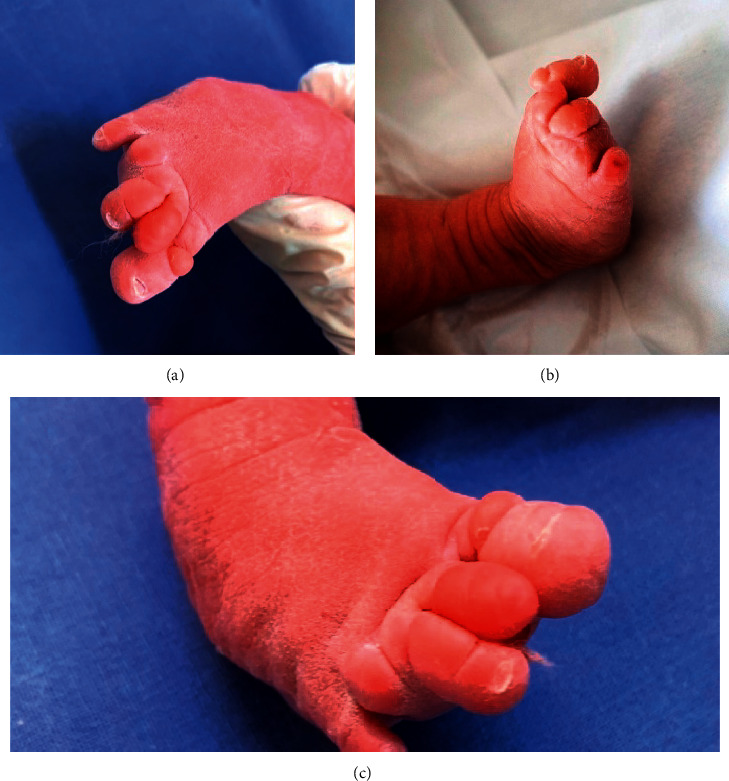
Right foot. Clubfoot on the right (a); syndactyly between 2 and 3^rd^ toe (b); macrodactyly of the big toe and partial amputation of the fourth toe (c).

The male newborn is the second child of related parents (first cousins) originating from a region in Kosovo. He was born at 36 weeks of gestation by caesarean section due to breech presentation and premature rupture of the amniotic membrane. His birth weight was 2.29 g (40^th^ percentile), his height was 43.5 cm (8^th^ percentile), and his head circumference was 31 cm (12^th^ percentile). The Apgar score at birth was 10 at both the 1^st^ and 5^th^ minute of life. Adaptation to postnatal life was good, with vigorous crying. Upon birth, a scaling skin and several malformations localized exclusively to the extremities were reported. The following malformations were observed: on the left hand, a deep single palmar crease, a deep annular sulcus on the fourth finger, and interdigital bridges (Figures 1(a)–1(c)); on the right hand, deep annular furrows on the third finger, spontaneous amputation of the fourth finger during the first hours of neonatal life, and presence of interdigital furrows (Figures 2(a)–2(c)); on the right foot, a clubfoot with a deep annular sulcus on the first toe, syndactyly between the 2^nd^ and 3^rd^ toe, macrodactyly of the allux, and partial amputation of the 4^th^ toe (Figures 3(a)–3(c)). The heart, lungs, hypochondriac organs, and genitals organs were normal. No facial dysmorphisms were found. Crying, eye contact, muscle tonus, and archaic reflexes that are normally present in clinical investigations for infectious diseases in the mother and in the proband were negative.

## 3. Discussion

ABS is a set of congenital malformations that mainly affect the limbs and, more rarely, the skull, face, chest, and abdomen. They are caused by the presence of bridles or bands stretched between the walls of the amniotic sac where extremities, limbs, or other parts of the fetus can “get caught,” undergoing constrictions and ischemia that lead to amputations and malformations that are typically polymorphic and asymmetric.

In the upper and lower limbs, the bridles can cause constriction with reduced blood flow resulting in partial or complete amputation of the limb. The severity and nature of the injuries can vary widely, ranging from cutaneous furrows of variable depth, to anomalies such as clubfoot, to complete limb amputation. However, limb agenesis, which can affect the upper or lower limbs, is extremely rare; transverse defects are more common. When ABS affects the skull, face, chest, or abdomen, highly variable and severe malformations can be observed that are usually fatal.

The cause of ABS is unclear, but is believed to arise from intrinsic or extrinsic factors. The intrinsic hypothesis suggests the development of constriction bands secondary to impaired blood flow with consequent damage to the fetus [3]. In support of this hypothesis, cases of ABS have been reported in which an intact amniotic sac was found. Moreover, this hypothesis may also explain why the involvement of internal organs can also be observed in subjects with ABS. The extrinsic hypothesis, however, suggests that ABS may arise when the inner layer of the amniotic sac is damaged, resulting in the formation of thin strands of fibrous tissue. These fiber-like bands of tissue can enwrap or constrict the body area involved, causing disruption of normal development [3–5]. The more widely accepted extrinsic model, proposed by Torpin, suggests a sequence mechanism of disruption of the amnion that leads to the entanglement of fetal structures by mesodermic bands [6]. Possible interference of genetic factors has also been proposed.

Although no commonly accepted risk factors have been identified, a retrospective cohort study found that the prevalence of ABS in people affected by vascular Ehlers–Danlos syndrome (vEDS) was significantly higher than that found in the general population. In this study, the prevalence of ABS in the general US population was given as 7 in 10,000 (0.07%), based on CDC data, whereas the prevalence in a cohort of vEDS patients was found to be 5 in 1,231 (0.4%) [7]. These data suggested that those with vEDS hold a five-fold greater risk of developing ABS relative to a control population [8]. Moreover, the recent case of two step-siblings who presented with ABS at birth was reported. The children had different mothers and shared a father who was suffering from vEDS. A variant of the *COL3A1* gene is documented in vEDS in which lysine is replaced by glutamic acid; the result is a reduced production of collagen type III, which causes fragility of the amniotic membrane [8].

Guzman-Huerta et al. [9] proposed to classify the ABS phenotypes into four groups: (1) craniofacial defect + limb defects; (2) craniofacial defect + limb defects + abdominal wall, spinal column, and/or thoracic defects; (3) limb defect + abdominal wall, spinal column, and/or thoracic defects; and (4) isolated defect (craniofacial, limbs, or thoracoabdominal wall). According to this classification, our case would fall into the fourth group.

Other than the characteristic limb anomalies observed in subjects with ABS, similar anomalies may recognize other etiological factors presenting as isolated or as part of a known syndrome. In such cases, anomalies of the upper and/or lower limbs may be present in individuals presenting with other deformities, including cleft palate, cleft lip, polydactyly, or severe heart or renal impairment [3]. Anomalies involving the extremities may be also one of the features of the Adams–Oliver syndrome (AOS), an inherited disorder presenting with abnormalities of the scalp and malformations of the hands, feet, and/or legs [3, 10–12].

The diagnosis of ABS is clinically determined, although it is also worthwhile to perform laboratory diagnostics. A histopathologic exam may reveal the absence of the amniotic membrane on the fetal surface of the chorionic sac (including the placenta); imaging studies could help to establish the location, type, and extent of the anomaly. Instead, genetic evaluation in the setting of ABS is not typically recommended, and although a few familial cases have been reported, almost all cases are sporadic with no identified underlying genetic cause. If ABS is suspected, chromosomal microarray analysis may be considered to rule out other possible causes [13].

ABS requires a multidisciplinary approach for treatment. Usually, surgery is performed, such as an elective and cosmetic surgery for patients with superficial bands that do not affect the lymphatic or circulatory drainage, or an emerging treatment is used for patients with deep bands affecting the anatomical and functional integrity of the site involved.

Prognosis depends on the time of diagnosis (most cases are diagnosed postnatally), type, and location of anomalies and may vary from cosmetic to life-threatening consequences [13]. In the case of postnatal diagnosis of anomalies exclusively localized to the limbs, as happened in our proband, the prognosis is good following surgical repair.

In conclusion, ABS is a rare but notable clinical concern. Future studies must be undertaken to further explore the most effective methods to prevent and treat this disorder and its associated complications.

## Data Availability

The data used to support the findings of this study are available from the corresponding author upon request.
